# Complete mitochondrial genome and the phylogenetic position of the Caroun croaker *Johnius carouna* (perciformes: Sciaenidae)

**DOI:** 10.1080/23802359.2018.1452648

**Published:** 2018-03-21

**Authors:** Wei-Di Yang, Chang-Chang Guo, Min Liu, Baian Lin

**Affiliations:** College of Ocean and Earth Sciences, Xiamen University, Xiamen City, Fujian Province, China

**Keywords:** *Johnius*, Bayesian tree, mitogenomes, phylogenetic relationship, Sciaenidae

## Abstract

In this study, the complete mitogenome of the Caroun croaker *Johnius carouna* was obtained. Its mitogenome is 18,752 bp in length, consisting of 37 genes with the typical gene order and direction of transcription in vertebrates. Gene rearrangement was found in *J. carouna*, same as another two *Johnius* species available in GenBank, *J. distinctus* (MF083699) and *J. grypotus* (KC491206). The overall nucleotide composition is: 24.2% A; 18.0% C; 21.4% G, and 36.4% T. Sizes of the 23 tRNA genes range from 67 to 75 bp. One start codons (ATG) and three stop codons (TAG, AGG, and TAA/TA/T) were detected in 13 protein-coding genes. In the Bayesian tree based on the complete mitogenomes of 21 species (including *J. carouna*) from the family Sciaenidae, all nodes were strongly supported. The result shows that *J. carouna* was placed as sister to the silver croaker *J. grypotus* of the same genus. The mechanism of gene rearrangement in the genus *Johnius* merits further investigation.

The family Sciaenidae (Perciformes) is commonly known as croakers and drums. As a commercially important group of fishes, the family comprises approximately 270 species in 70 genera in the world (Nelson 2006). The genus *Johnius* consists of about 30 small to medium-sized species endemic to Indo-West Pacific. The Caroun croaker *Johnius carouna* (Cuvier, 1830) inhabits along shallow coastal waters and enters estuaries and mangrove swamps, found from the north to southern China to the West India (Sasaki 2001). In this study, we presented the complete mitochondrial genome of *J. carouna* and analyzed its phylogenetic relationship based on another 20 available mitogenomes in Sciaenidae using one available mitogenome in the family Epinephelidae and two in the family Polynemidae as an outgroup.

One specimen of *J. carouna* was collected by gill net in the coastal water of Naozhou Island, Guangdong Province, China. The protocol and data analysis methods followed Chen et al. (2014). The complete mitochondrial genome of *J. carouna* is 18,752 bp in length (GenBank accession number: MF004312) with the typical gene order and transcriptional direction in vertebrates and with gene rearrangement feature. It contains two rRNA genes, 23 tRNA genes, 13 protein-coding genes, and 6 major noncoding regions including one control region and 5 other non-coding regions named as NC1 to NC5. The overall nucleotide composition is as follows: 24.2% A; 18.0% C; 21.4% G, and 36.4% T. In the 13 protein-coding genes, only one start codon (ATG) was detected. Three stop codons (TAG, AGG, and TAA/TA/T) were found; *ND1*, *ND5,* and *ND6* was terminated by the TAG codon, *COX1* by the AGG codon, and the other 9 protein-coding genes by either the TAA or incomplete T or TA codon that may form the complete termination signal UAA via post-transcriptional polyadenylation (Ojala et al. 1981). The 12S (962 bp) and 16S (1701 bp) rRNA genes are located between the tRNA-*Phe* and tRNA-*Leu1* genes, separated by a 65 bp non-coding region. The lengths of 23 tRNA genes range from 67 to 75 bp without any inserted sequences were identified as the putative origin of L-strand replication (OL). The control region was 1035 bp in length with high A + T (65.3%) and low G + C (34.7%) composition, located between the tRNA-*Val* and tRNA-*Phe* genes.

Published mitogenomes of all 21 species from Sciaenidae (including *J. carouna* in this study) together with *Cephalopholis boenak* from Epinephelidae, and *Eleutheronema tetradactylum* and *Polydactylus sextarius* from Polynemidae were used to assess the phylogenetic relationship of *J. carouna*. Phylogenetic tree was constructed with the partitioned Bayesian method based on the dataset combined by three partitions under the GRT + I + G model (Ronquist and Huelsenbeck 2003). As the phylogenetic tree showed, all nodes were strongly supported with high value of posterior probability (Figure 1). The result showed that *J. carouna* was placed as sister to the *J. grypotus* of the same genus. More mitogenomes from the genus *Johnius*, and the mechanism of gene rearrangement in this genus merit further investigation.

**Figure 1.**
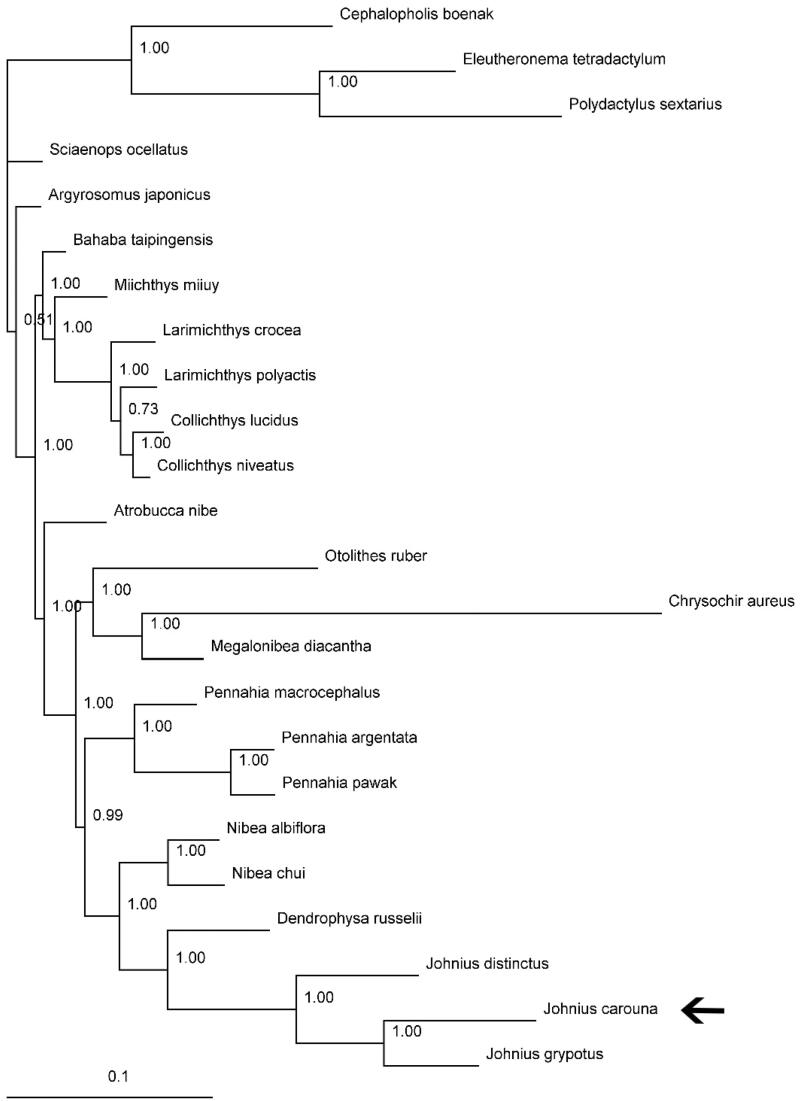
Phylogenetic position of the Caroun croaker *J. carouna* (MF004312). *Cephalopholis boenak* (KC537759), *Eleutheronema tetradactylum* (KC878730) and *Polydactylus sextarius* (NC_027088) were selected as the outgroup. The other 20 species from Sciaenidae are: *Argyrosomus japonicus* (KT184692), *Atrobucca nibe* (MF004314), *Bahaba taipingensis* (NC_018347), *Chrysochir aureus* (MF004313), *Collichthys lucidus* (JN857362), *Collichthys niveatus* (JN678726), *Dendrophysa russelii* (JQ728562), *Johnius distinctus* (MF083699), *Johnius grypotus* (KC491206), *Larimichthys crocea* (NC_011710), *Larimichthys polyactis* (GU586227), *Megalonibea diacantha* (KM257722), *Miichthys miiuy* (NC_014351), *Nibea albiflora* (NC_015205), *Nibea chui* (NC_025307), *Otolithes ruber* (KX929060), *Pennahia argentata* (NC_015202), *Pennahia macrocephalus* (KX576460), *Pennahia pawak* (KY978753) and *Sciaenops ocellatus* (NC_016867).

## Sample collection and DNA storage

The samples of *J. carouna* were collected in the coastal water of Naozhou Island in the South China Sea.

The samples and DNA of *J. carouna* were stored in the Fish Biology Laboratory in Xiamen University, China.
